# Concurrent chemoradiotherapy with low dose weekly gemcitabine in stage III non-small cell lung cancer

**DOI:** 10.1186/1471-2407-5-71

**Published:** 2005-07-06

**Authors:** Ufuk Abacioglu, Perran F Yumuk, Hale Caglar, Meric Sengoz, Nazim S Turhal

**Affiliations:** 1 Department of Radiation Oncology, Marmara University Medical School Hospital, Istanbul, Turkey; 2 Division of Medical Oncology, Marmara University Medical School Hospital, Istanbul, Turkey

## Abstract

**Background:**

Combined chemoradiotherapy (CRT) is the treatment of choice for stage III NSCLC. Gemcitabine (G) is a novel deoxycitidine analogue that has been proven to be a potent radiosensitizer. Twenty-two consecutive patients were treated with concurrent CRT to demonstrate the tolerability and efficacy of low dose G given weekly as radiosensitizer in stage III NSCLC.

**Methods:**

Patients with KPS ≥70, adequate bone marrow reserve, with no prior radiotherapy (RT) and surgery were included. Eighteen patients had received prior induction chemotherapy (CT). G (75 mg/m^2^/week) was infused over 1 hour for 6 weeks. Thoracic RT was given two hours later over 6 weeks at 1.8 Gy/day fractions (total dose of 61.2 Gy). Pulmonary toxicity was evaluated with computed tomography scans in 6 weeks.

**Results:**

Median age was 60 years (range, 48–75), median follow-up was 15 months (range, 2–40). Sixty-eight percent of patients were male and median KPS score was 90. Conformal 3D-RT planning was used in 64% of patients. G was given for a median of 5 weeks (range 1–9). Twelve patients (54.6%) received all planned CT. G was stopped because of intolerance in 6 and death in 2 patients. Seven patients (31.8%) had radiation pneumonitis. Twenty patients were evaluated for overall response, 1 patient (4.5%) had clinical CR, 81.8% had PR while 9.5% had SD. Median overall survival (OS) was 14 ± 5 months (95% CI 3–25). One- and 2-year OS rates were 55% and 38%. Sixteen patients died of disease-related events (6 with progression of primary tumor, 8 due to metastatic disease), 2 patients died of other causes. One- and 2-year progression-free survival and local control rates were 56%, 27% and 79%, 51%, respectively.

**Conclusion:**

G might be used as radiosensitizer for patients with stage III NSCLC who could not receive full doses CT with concurrent RT.

## Background

Lung cancer is the leading cause of cancer related deaths all around the world. About 80% of all lung cancer patients are non-small cell lung cancer (NSCLC) and 20–30% of these patients present with locally advanced disease. Although 1-year survival rate of patients with local disease is more than 70%, this rate is about 40% for patients with stage III disease [1]. Chemoradiotherapy (CRT) is considered the gold standard treatment of these patients after significant survival advantage and increase in local control had been shown by 3 meta-analyses [2-4]. Sequential versus concurrent CRT has been further investigated and concurrent CRT has been found to be superior [5-7]. Despite advances in treatment modalities overall 5-year survival rate for locally advanced NSCLC remains less than 15% [8].

Gemcitabine (G) is a deoxycytidine analogue that demonstrated activity in NSCLC as single agent and in combination also has been proven to be a potent radiosensitizer. Mechanism of radiosensitizing activity of G is not well understood. Possible mechanisms that have been shown in preclinical studies are reduction of apoptotic threshold for radiation due to intracellular dATP pool depletion with simultaneous redistribution of cells into the S phase [9]. As a result of these factors DNA damage caused by radiation cannot be properly repaired, apoptosis was induced and cell death increased. The average enhancement ratio of G is ≥1.5 and sensitization persists for at least 72 hours [10]. Preclinical data supports that more frequent G dosing schedule with radiation could yield local control advantages in the setting of a clinical trial [11].

In this study it is aimed to demonstrate the efficacy and tolerability of low dose G given weekly as radiosensitizer in stage III NSCLC patients.

## Methods

Consecutive patients between the ages 18–75 years with histological or cytological confirmed stage III NSCLC who had Karnofsky Performance Status (KPS) ≥70 in Medical and Radiation Oncology Clinics of Marmara University between December 1999 and July 2002 were considered for this study. Other patient selection criteria were white blood cell count ≥3000/mm^3^, hemoglobin level ≥10 gr/dl, platelet count ≥100000/mm^3^, no prior RT or lung resection accept open lung biopsy or mediastinoscopy for diagnostic purposes. All combinations and cycles of induction CT prior to planned concurrent CRT were eligible as the ones who did not receive any induction CT. All patients started CRT within 1 month after completion of induction CT. There was not a limitation to the lung volume planned to be irradiated.

The overall dose of RT was 60–61.2 Gy administered over 6 weeks with daily fractions of 2 Gy administered for 5 days each week. Planning computed tomography scan (CTS) was performed for each patient in supine position. The planning target volume (PTV) consisted of radiological visible primary tumor with a margin of 2 cm in each diameter. Elective nodal irradiation field included the PTV for the first 6 patients. After 6 patients the treatment planning and volume was amended to multiple field arrangements with custom blocking to all fields and involved hilar and mediastinal lymph nodes. Irradiation was administered with megavoltage photons of at least 6 MV. The dose of 60 Gy was specified at the isocenter of the central axis and was corrected for pulmonary heterogeneity. The spinal cord was not allowed to receive RT more than 45 Gy.

G (75 mg/m^2^/week) was infused each Monday by i.v. route over 1 hour in 500 cc of 0.9% normal saline for 6 weeks. RT was administered 2 hours after the G infusion. 5-HT antagonists were given 30 minutes prior to G infusion.

Patients were followed weekly with routine physical examination and complete blood count (CBC). Toxicity was graded according to the National Cancer Institute Common Toxicity Criteria (CTC), version 2.0 [12]. If the patient had WBC ≤2500/mm^3^, platelets ≤50000/mm^3 ^or grade 3 or 4 hematological toxicity, G was not administered that week. G was stopped if CBC was as low as mentioned above for 2 consecutive weeks or grade ≥3 non-hematological toxicities were seen during the treatment period.

Pulmonary toxicity was evaluated with CTS of the thorax 6 weeks after the completion of RT. Patients having ground glass appearance or alveolar consolidation on CTS were started on corticosteroids (40 mg of fluocortolon daily, which is the standard treatment protocol of the centre) for 2–4 weeks even if they were asymptomatic. Responses were routinely evaluated in 3 months. Standard World Health Organization (WHO) criteria were used to determine response [13]. Tumor volume at the beginning of CRT was compared to the volume at the end of this treatment and this difference was used to calculate the response rate. Survival rates were calculated by using the Kaplan-Meier method.

## Results

Total of 22 consecutive patients were treated with this treatment protocol. Patient characteristics are given in Table 1. Median age was 60 years (range, 48–75) and 68.2% were male. Median KPS score was 90 (range, 70–100). This group had a median 43 pack-years smoking history (range, 0–90). Fifty percent of the patients had stage IIIA disease. Twelve patients (54.5%) had squamous cell histology. Eighteen patients had induction CT and median number of cycles was 3 (range, 2–8). One patient received 8 cycles, 3 patients received 6 cycles, 3 patients received 4 cycles of induction CT. After induction CT, 9 patients (40.9%) had partial response (PR), 5 (22.7%) had stable disease (SD) and 4 (18.2%) had progressive disease (PD). The patient who had 8 cycles of induction CT received 4 cycles of two different CT regimens and had PD after both. Two of the other three patients who received 6 cycles of induction CT had SD and 1 had PR.

Conformal 3D-RT planning was used in 63.6% of the patients. Median RT dose administered was 60 Gy (range, 32–66 Gy) with 1.8 Gy (range, 1.8–2 Gy) daily fraction in a median of 46 days (range, 29–64 days) over 7 weeks (range, 4–9 weeks). Elective nodal irradiation was given in 6 patients (27.3%). Rest of the patients (72.7%) received RT only to tumor and involved hilar and mediastinal lymph nodes (Table 2).

G was given for a median of 5 weeks (range, 1–9). Twelve patients (54.6%) received all planned CT. G was stopped in 8 patients, because of intolerance (grade 2–3 fatigue, nausea, vomiting, esophagitis and low CBC for 2 consecutive weeks) in 6 and death during treatment in 2. One patient who died of pneumonia had asymptomatic congestive heart failure and 52.5 pack-years smoking history. Another patient died with acute onset of loss of consciousness, hypotension, breathing depression, and this was considered to be secondary to cerebrovascular accident. Both deaths were seen on the 5th week of treatment. In 2 patients G was held in the middle of the treatment for 1 week because of grade 2 hematological toxicity. Neutropenia and anemia were the most significant toxicities observed (Table 2 and 3). There were only 4 patients (18.2%) who had acute grade 3 esophagitis, while no other grade 3 acute toxicity was seen. These 3 patients had recovered completely after completion of RT. Seven patients (31.8 %) had radiation pneumonitis; four (18.2%) of these had grade 1, two (9.1 %) had grade 2 and one patient had grade 5 (4.5%) (Table 3). One other patient was not assessed because of new brain metastasis was seen right after the completion of her CRT. All patients with signs of radiation pneumonitis on their CTS received corticosteroid treatment, according to our centers' protocol.

One patient (4.5%) had clinical CR, 18 (81.8%) had PR, while 2 (9.5%) had SD after CRT. Two patients who had PR were operated (1 had lobectomy, the other pneumonectomy). Two other patients received adjuvant CT after completion of CRT (Table 3).

**Figure 1:**
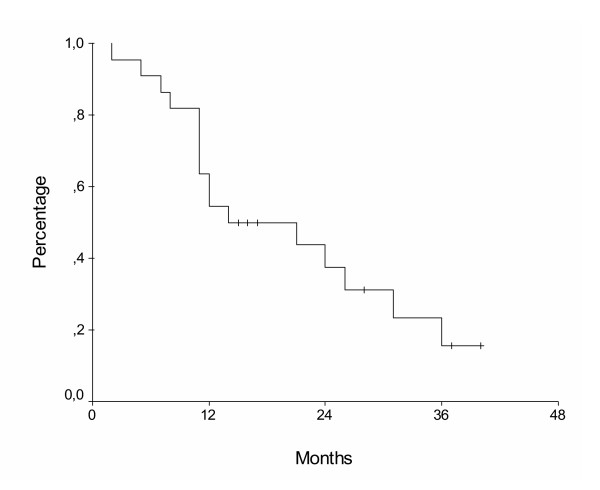
Overall survival plot for all patients.

Median follow-up was 15 months (range, 2–40 months). Median overall survival (OS) was 14 months (Figure 1). One- and 2-year OS rates were 54.5% and 37.5%. Sixteen patients (72.7%) died, 6 (27.3%) with progression of their primary NSCLC, 8 due to metastatic disease (brain metastasis in 6, contralateral lung metastasis in 2), 1 with pneumonia and another with cerebrovascular event. Median disease free survival (DFS) was 14 months, while 1- and 2-year DFS rates were 55.5% and 26.5%, respectively. Seven patients (31.8%) progressed locally, while 8 other (36.4%) had distant metastasis during follow-up after CRT. Another patient had both local progression and distant metastasis. Five of the patients with local progression had stage IIIB disease initially. First site of metastasis seen in this group was brain in 4, contralateral lung in 3, skin in 1 and neck in another patient. Local control was achieved in 63.6% of the patients. One- and 2-year local control rates were 78.6% and 51%, respectively (Table 4).

## Discussion

For many years the mainstay of treatment for non-resectable NSCLC was RT alone. The results of several phase II studies provided support for the addition of CT to RT and a landmark trial was reported by Dillman et al in 1990 [14]. He demonstrated increased rates of three-year and long-term survival [15]. Two large meta-analyses have provided support for the benefit of CRT combinations [3,4].

The initial phase I and II clinical experience in NSCLC had demonstrated that weekly G (600–1000 mg/m^2^) with concurrent standard thoracic RT was effective, but it had significant toxicity (pulmonary fibrosis, esophagitis, thrombocytopenia and neutropenia) [16-21]. Doses of G used in these preliminary phase II studies were not derived from well-planned phase I studies and were very high. Also significant number of patients had dose reductions or they missed doses during the treatment. After these unclear results dose escalating trials of G as a radiosensitizer with concurrent RT were started [22,23]. In one of the first studies Gonzalez et al recommended that G can be used at 300 mg/m^2^/week dose with 50 Gy of RT, but the dose of RT was lower than standard in this trial [16]. McMullen et al reported in their phase I/II trial (CALGB 89805) that 70 mg/m^2^/week was the recommended dose of G with 60–74 Gy RT [23]. Trodella et al recommended weekly G dose of 350 mg/m^2 ^for 5 weeks concurrent with 50.4 Gy RT after their phase I trial [24].

Our rationale for evaluating weekly 75 mg/m^2 ^dosing was based upon a phase I study of Blackstock et al [22]. In that study they suggested that thoracic RT and G given with a dose of weekly 70 mg/m^2 ^in two days appeared tolerable in patients with advanced NSCLC. Although this was a phase I study, overall response rate for 16 patients was 88% and median survival for all 17 patients was 16 months. Dose limiting toxicities were pulmonary (pneumonitis, pulmonary fibrosis), esophageal (esophagitis, esophageal stricture, ulceration), and hematological (anemia, thrombocytopenia) toxicities in this CALGB trial [22]. They delivered almost all the planned G doses (range, 7–12). Several preclinical [25-27] and clinical [28,29] trials have also shown G to be an effective RT enhancer, even when low doses are administered.

In this study the RT planning was changed from 2D conventional to 3D conformal multiple fields planning after the first 6 patients. G given concurrently with 3D-RT is much better tolerated than with 2D-RT and a reduction in grade 3 esophagitis was seen, as it was reported by Zinner et al [30]. The maximal tolerated dose of G given concurrently 7 weeks on row with RT as a radiosensitizer was 190 mg/m^2^/week with 3D planning while 125 mg/m^2^/week could be administered with 2D-RT in that study. In our study the differences in the rates of grade 3 or 4 esophagitis and pneumonia between the two treatment schedules were not significant. This might be due to having small number of patients in our study. We also changed elective nodal irradiation to involved nodal irradiation after the first 6 patients. Emami et al concluded in their analysis of the RTOG data that elective irradiation of uninvolved supraclavicular and mediastinal nodes may not be necessary in the treatment of unresectable NSCLC [31].

Blackstock et al also reported that there appeared to be a relationship between volume of lung irradiated, pre-treatment pulmonary function and pulmonary fibrosis, although the data was limited in their phase I trial [22]. Pre-treatment volumes of the tissue irradiated was not calculated in our patient population, but they were much larger than 2000 cm^3 ^which was reported as a cut off in inclusion criteria in the trial reported by Price et al. [28]. Therefore we favored to administer a much lower dose of G then what was used in the above trial.

G and cisplatin have also been studied with concurrent RT. Phase II data have been reported during the last years. Vokes et al recommended 600 mg/m^2^/day, day 1 and 8 of G and 80 mg/m^2^/day of cisplatin repeated every 21 days × 2 courses during RT (66 Gy) and this was the dose used in CALGB 9431 [32]. There was a marked increase in the toxicities of this schedule, especially in hematological toxicities and esophagitis. Although this was a phase II study, median survival was reported as 18.3 months which is 1–2 months longer than other concurrent CRT studies. In several randomized phase III trials comparing chemotherapy (either sequential or concomitant) with RT alone, the median survival was found between 8–18 months [3]. In our study median survival was 14 months. Although the survival data's extracted from phase II studies should not be used for comparison, this survival difference between our pilot study and other phase II data might be secondary to using lower dose of G in our study, having 2 very early deaths, lower dose of RT administered, not using cisplatin, treating patients with more tumor load or nature of the disease. But the median survival in our study was similar to the phase III data [3].

In our study there might be two reasons for the high incidence of radiation pneumonitis. The first reason might be the high number of cycles of induction CT. Three of the four patients receiving 6 or more cycles of induction had grade 1 or 2 pneumonitis. Although chemotherapeautic agents were found to induce radiation pneumonitis either alone or with radiation therapy in previous studies this was not seen in our patients [33-35]. The other reason might be the screening schedule of pneumonitis in our centre. All of the patients were considered for radiation pneumonitis during the first CTS examination for the tumor response. The patients received corticosteroid therapy for radiation pneumonitis according to the CTS images but most of them were asymptomatic.

The patients could not receive all planned G dose concurrent with RT. Heavily pre-treated patients with induction CT before CRT might have not tolerated weekly planned G schedule. Eighteen of the patients had induction CT, the median number of cycles were 3 and seven of the patients received ≥4 cycles. The reasons for this were that patients either progressed during induction CT and received second line CT regimens or longer induction CT was their physicians decision before referring them to our centre for RT.

## Conclusion

G might be used as radiosensitizer for patients with stage III NSCLC who could not receive full doses CT with concurrent RT. In order to decrease the therapy associated toxicities smaller irradiation volumes used with 3D conformal techniques might be better. We need prospective randomized trials with increased number of subjects in order to figure out where this regimen exactly stands in the standard treatment of this group of patients.

## Competing interests

The author(s) declare that they have no competing interests.

## Authors' contributions

UA, PFY, HC, MS, NST designed the study, followed the patients, collected the data, performed the statistical analysis and drafted the manuscript. All authors read and approved the final manuscript.

## Pre-publication history

The pre-publication history for this paper can be accessed here:



## Figures and Tables

**Table 1:** Patient characteristics

Characteristics	Number (n)	Percentage (%)
Gender		
Male	15	68.2
Female	7	31.8

Stage		
IIIA	11	50
IIIB	11	50

KPS		
70	4	18.2
80	5	22.7
90	12	54.5
100	1	4.5

Histology		
Adenocarcinoma	6	27.3
Squamous cell carcinoma	12	54.5
Adenosquamous cell carcinoma	1	4.5
Unclassified	3	13.6

ICT		
Yes	18	81.8
No	4	18.2

ICT Regimens		
PE	4	22.2
NP or NC	6	33.3
GP	3	16.7
INC	2	11.1
TC	2	11.1
INC + TC	1	5.6

Number of ICT Cycles		
2–3	11	61.1
≥4	7	38.9

Response to ICT		
PR	9	50
SD	5	27.8
PD	4	22.2

ICT: Induction Chemotherapy, PE: Cisplatin-Etoposide, NP: Vinorelbine-Cisplatin, NC: Vinorelbine-Carboplatin, GP: Gemcitabine-Cisplatin, INC: Ifosfamide-Navelbine-Carboplatin, TC: Paclitaxel-Carboplatin, PR: Partial Response, SD: Stable Disease, PD: Progressive Disease

**Table 2:** Treatment characteristics of the patients

Variable	Number (n)	Percentage (%)
RT Planning		
Conventional	8	36.4
Conformal	14	63.6

RT Dose/Fraction		
1.8 Gy	13	59.1
2 Gy	9	40.9

Number of RT Treatment Weeks		
4	1	4.5
5–7	16	72.7
≥8	5	22.7

Number of G Treatment Weeks		
≤4	7	31.8
5–7	13	59.1
≥8	2	9.1

RT: Radiotherapy, G: Gemcitabine

**Table 3:** Non-hematological and hematological toxicities for CRT and responses

Variable	Number (n)	Percentage (%)
Pneumonia	7	31.8
Grade 1	4	18.2
Grade 2	2	9.1
Grade 5	1	4.5

Nausea/Vomiting	4	18.2
Grade 1	2	9.1
Grade 2	2	9.1

Esophagitis	16	72.8
Grade 1	8	36.4
Grade 2	4	18.2
Grade 3	4	18.2

Hematological toxicity	5	22.7
Grade 1	3	13.6
Grade 2	2	9.1

Response to CRT		
Clinical CR	1	4.5
PR	18	81.8
SD	2	9.1
Unknown	1	4.5

CRT: Chemoradiotherapy, CR: Complete Response, PR: Partial Response, SD: Stable disease

**Table 4:** Overall survival, progression-free survival and local control data

	Months ± SD (95% Confidence Interval)	
Median OS	14 ± 5 (3–25)	
Median PFS	14 ± 3 (8–20)	
	1-year (%)	2-year (%)
OS Rates	55	38
PFS Rates	56	27
Local Control Rates	79	51

OS: Overall survival, PFS: Progression-free survival
